# Single Nematode Transcriptomic Analysis, Using Long-Read Technology, Reveals Two Novel Virulence Gene Candidates in the Soybean Cyst Nematode, *Heterodera glycines*

**DOI:** 10.3390/ijms24119440

**Published:** 2023-05-29

**Authors:** Dave T. Ste-Croix, Richard R. Bélanger, Benjamin Mimee

**Affiliations:** 1 Saint-Jean-sur-Richelieu Research and Development Centre, Agriculture and Agri-Food Canada, Saint-Jean-sur-Richelieu, QC J3B 3E6, Canada; dave.thibouthotste-croix@agr.gc.ca; 2 Département de Phytologie, Université Laval, Québec, QC G1V 0A6, Canada; richard.belanger@fsaa.ulaval.ca; 3 Centre de Recherche et d’Innovation sur les Végétaux (CRIV), Université Laval, Québec, QC G1V 0A6, Canada

**Keywords:** *Heterodera glycines*, SCN, virulence, MinION, single nematode, effectors, alternative splicing

## Abstract

The soybean cyst nematode (*Heterodera glycines*, SCN), is the most damaging disease of soybean in North America. While management of this pest using resistant soybean is generally still effective, prolonged exposure to cultivars derived from the same source of resistance (PI 88788) has led to the emergence of virulence. Currently, the underlying mechanisms responsible for resistance breakdown remain unknown. In this study, we combined a single nematode transcriptomic profiling approach with long-read sequencing to reannotate the SCN genome. This resulted in the annotation of 1932 novel transcripts and 281 novel gene features. Using a transcript-level quantification approach, we identified eight novel effector candidates overexpressed in PI 88788 virulent nematodes in the late infection stage. Among these were the novel gene Hg-CPZ-1 and a pioneer effector transcript generated through the alternative splicing of the non-effector gene Hetgly21698. While our results demonstrate that alternative splicing in effectors does occur, we found limited evidence of direct involvement in the breakdown of resistance. However, our analysis highlighted a distinct pattern of effector upregulation in response to PI 88788 resistance indicative of a possible adaptation process by SCN to host resistance.

## 1. Introduction

The soybean cyst nematode (*Heterodera glycines Ichinohe*, SCN), is the single most damaging pest to soybean production, causing over USD 1.2 billion in yield losses annually in North America [1,2]. Successful parasitism by this biotrophic plant-parasite is dependent on the establishment of a specialized feeding structure, called the syncytium. This complex reorganization of soybean tissues into a biologically active feeding site is mediated through the coordinated secretion of many effector proteins [i.e., small nematode-derived and stylet-secreted proteins] [3]. In SCN, effectors are mainly produced in a set of specialized esophageal glands, notably one dorsal and two subventral glands, and are involved in many key parasitic functions. These include cell-wall degradation, plant hormone system trafficking (through hormone-mimicking proteins), cellular transcriptional reprogramming, and suppression of the host immune/stress response [4,5]. To date, over 80 distinct effector genes involved in the early stages of parasitism have been documented through gland-cell targeted cDNA sequencing and hybridization studies [6,7,8,9,10]. While these gland-cell targeted studies have proven valuable, effectors secreted in other nematode tissues [such as in amphids or on the cuticle surface] [11], released through unconventional protein secretion (UPS) pathways or expressed in later stages of SCN parasitism have received limited attention.

In North America, crop rotations, used alongside SCN-resistant soybeans, remain the most effective method for controlling SCN populations [12,13]. Currently, just over 95% of all commercially available resistant varieties are derived from a single source of resistance found in the plant introduction (PI) accession 88788 (PI 88788) [12]. Resistance in this PI is conferred by a high copy number of the resistant allele *rhg1-b*: a 31kb genomic segment harboring three dissimilar genes including an amino acid transporter (GmAAT), an α-soluble N-ethylmaleimide-sensitive factor (NSF) attachment protein (GmSNAP18) variant and a wound-inducible protein (GmWI12) [14]. While still effective, the widespread and systematic use of this limited source of resistance has resulted in a strong selective pressure leading to the emergence of subpopulations of SCN able to overcome host resistance. For example, a recent study across Missouri found that PI 88788-type resistance was overcome in all 393 fields sampled by the authors; with similar findings reported in Illinois where virulence was detected in 109 out of the 156 fields sampled [15,16]. Yet, to date, little is known regarding the identity of SCN effectors involved in the breakdown of host resistance with most candidate virulence genes identified thus far lacking functional evidence and/or validation in field populations [17,18,19].

In the SCN-soybean pathosystem, two hypotheses may explain how virulence is acquired. First, differences in gene expression [stemming from regulation, epigenetic control or differences in gene copy number], could result in the repression of an *Avr* protein or in the overproduction of a virulence factor [a protein capable of breaking down plant resistance]. Second, sequence polymorphism and/or structural variation within effector proteins [resulting from mutations or alternative splicing] could hinder host recognition, alter *Avr* gene transcript abundance or lead to new parasitic functions [20,21,22].

Traditionally, the discovery of gene splice isoforms has relied on short-reads, such as those produced by next-generation sequencing platforms. While plentiful, these reads rarely ever span across splice junctions, making the reconstruction of full-length transcripts challenging and prone to errors [23,24]. This is particularly true in cases where alternative splicing generates multiple partially redundant isoforms at a given locus [23]. However, in recent years, the continuing development of long-read sequencing technologies has made it possible to sequence the full length of transcripts in a single molecule, thereby eliminating the challenges posed by the computational assembly and delivering a reliable set of isoforms [23].

Although enormous progress has been made in the annotation of the SCN genome, the number of reported genes between assemblies of the same isolate, and between different chromosomal assemblies, vary considerably, emphasizing the likelihood that genes of interest may remain unreported [25,26,27]. Using a single-nematode transcriptomic approach, first applied in SCN to limit the challenges associated with the high degree of genetic diversity within populations [19], we sequenced the whole transcriptome of single J4-staged female nematodes developing on both susceptible and resistant soybean lines using the long-read sequencing capabilities of the ONT MinION. The reannotation of the SCN genome, through multiple single nematode long-read sequencing of transcriptomes, revealed multiple new isoforms as well as new gene models, including some that were differentially expressed in virulent individuals.

## 2. Results

### 2.1. MinION Sequencing Efficiency and Transcript Variants Discovery

**Figure 1:**
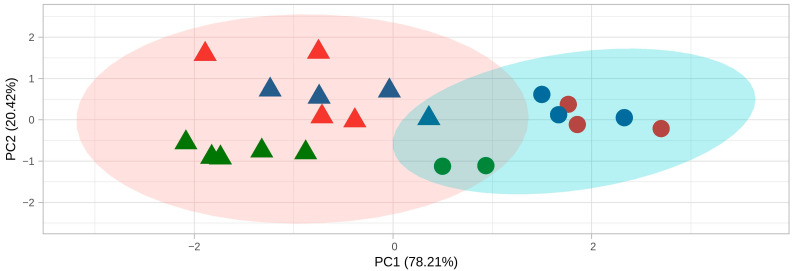
Post-filtering principal component analysis of the top 200 differentially expressed transcripts in individual *Heterodera glycines* nematodes. Nematodes collected from susceptible soybean are denoted as triangles while nematodes collected from the resistant soybean PI 88788 are represented as circles. The point color denotes the population of origin of the sample where Green is QCSTJ5, Blue is QCSTJ6, and Red is QCSTJ15. Expressional clusters are represented as colored ellipses where red represents the avirulent cluster and blue the PI88788-vir cluster.

Nanopore sequencing of three soybean cyst nematode populations (Table 1), differentially selected on soybean to select specific virulence phenotypes (workflow shown in Figure S1), generated a total of 54,403,200 long-read cDNA sequences. Population-wise, sequencing of QCSTJ15, QCSTJ5 and QCSTJ6 produced 21,764,670, 19,310,846 and 13,327,684 reads, respectively. The number of mapped reads per individual nematode varied considerably, ranging from 64,486 to 2,195,899 sequences with an average number of 677,167 sequences per sample. Of the 27 individual females sequenced, four were lost in sequencing due to low mapping rates (QCSTJ5_PI1, QCSTJ5_Ess5, QCSTJ15_Ess2, QCSTJ15_Ess6). Additionally, two samples collected from the susceptible Essex plants were removed due to clustering with PI88788-vir nematodes indicating expression similarities (QCSTJ6_Ess4 and QCSTJ6_Ess6) (Table S1). A principal component analysis (PCA) of the top 200 differentially expressed transcripts, across the remaining samples, highlighted two distinct clusters representing PI88788-vir and Avir nematodes, respectively (Figure 1). The population of origin of each individual also had a strong impact on PCA distribution. Samples from populations QCSTJ6 and QCSTJ15 were found to cluster more tightly together while samples from QCSTJ5 clustered separately. However, when the PCA analysis was restricted to nematodes stemming from the same population, PI88788-vir nematodes were found to consistently cluster together with Avir nematodes loosely clustered around.

### 2.2. Reannotation of the SCN Genome

Detection of novel transcript isoforms, using StringTie2 under stringent parameters, identified 1932 novel variants, expressed at a minimum threshold of 10 TPM, bringing the total number of SCN transcripts within our dataset to 25,865. The majority of these novel isoforms were located in known coding gene features of SCN (1622 isoforms); with the remainder located in putative novel coding gene features (310 isoforms in 281 gene features) (Table S2). To reduce the impact of redundant isoform models on transcript quantification, caused by gene duplication events, this set of transcripts was clustered into a set of 24,103 non-redundant models (File S1). Overall, 22,280 of these 24,103 transcripts were observed at least once in the present study.

### 2.3. Differential Transcript Expression Analysis

A differential expression analysis identified 171 differentially expressed transcripts with 35 stemming from novel transcript variants identified through StringTie2. Interestingly, all of these differentially expressed transcripts were up-regulated in PI88788-vir females (Table S3). Protein secretion analysis, either via SignalP or SecretomeP, identified 11 transcripts containing secretion signal peptides with an additional 20 containing features for non-classical protein secretion. None of these candidate effectors contained transmembrane domains as predicted by TMHMM-2.0. Protein subcellular localization, predicted by DeepLoc-2.0, revealed that only eight of these putative effectors were predicted to be localized to the extracellular region with other candidates predicted within the mitochondrion (*n* = 3), the cytoplasm (*n* = 7) and the nucleus (*n* = 6) among others (Table 2). Within differentially expressed transcripts identified as putative effectors, and predicted to be localized to the extracellular region, were three transcripts of unknown protein function (Hetgly02290.t1, Hetgly06908.t1 and Hetgly08731.t1), two transthyretin-like proteins (Hetgly.04491.t1; Hetgly21677.t1), a major allergen-like protein (Hetgly07480.t1) and two *Heterodera avenae* related putative effector proteins (Hg08859.1 and Hg16414.1). All but one (Hg16414.1) of these transcripts were located in gene features with only one known transcript. Analysis of the available gland-cell RNA-Seq data showed that three of these candidates (Hetgly.04491.t1, Hg8859.1, and Hg16414.1) were also expressed in parasitic J2-staged secretory glands.

**Figure 2:**
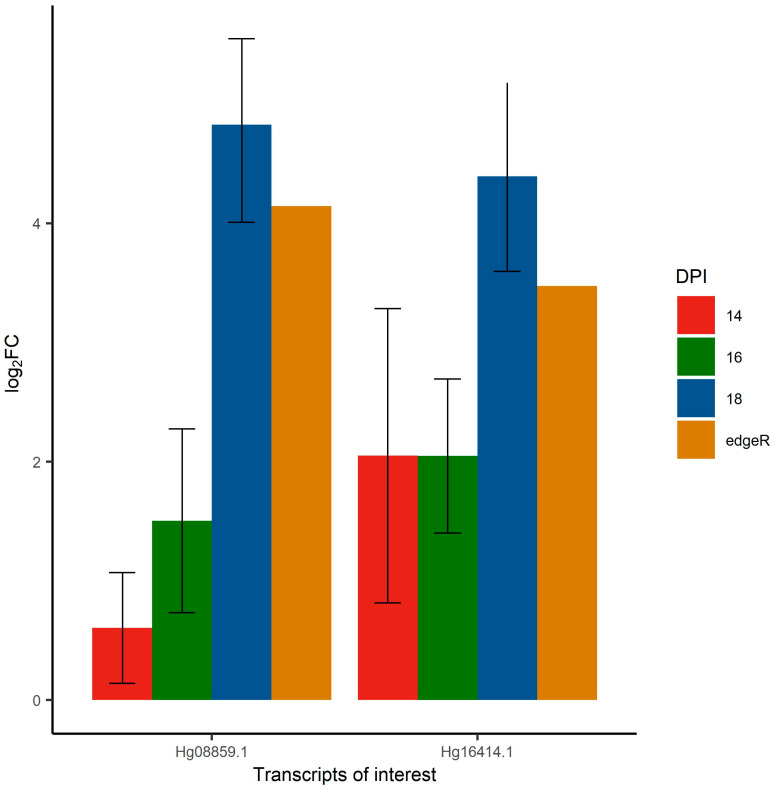
Relative fold change (log_2_FC) estimates of different transcripts of interest in *Heterodera glycines* PI 88788 virulent females at three different time points compared to avirulent females obtained by qRT-PCR. Error bars represent the standard error surrounding the relative log_2_FC estimate. The “edgeR” value represents log_2_FC estimate of the differential expression analysis based on sequencing reads using the edgeR tool.

In the context of this study, Hg08859.1 and Hg16414.1 were of particular interest and were further investigated. These candidates were found to be novel SCN transcripts consistently over-expressed in PI88788-vir females. On average, Hg08859.1 had a 4.145 log_2_FC while Hg16414.1 had a 3.474 log_2_FC compared to their expression in avirulent females. Validation of these targets, through qPCR SybrGreen-based assay, showed similar levels of differential expression at 18 DAI with relative log_2_FC estimates of 4.827 ± 0.819 and 4.394 ± 0.797 in Hg08859.1 and Hg16414.1, respectively. Relative log_2_FC estimates were lower in both transcripts at 16 and 14 DAI (Figure 2).

**Figure 3:**
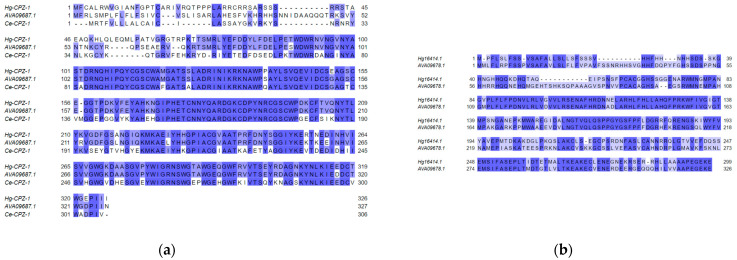
Multiple sequence alignment of candidate virulence gene (**a**) Hg-CPZ-1 with putative effector AVA09687.1 from *Heterodera avenae* and Ce-CPZ-1 from *C. elegans* and (**b**) Hg16414.1 with putative effector AVA09678.1 from *H. avenae*. Residue color represents the similarity between sequences at a given amino acid position.

Located on Chromosome 4, Hg08859.1 showed the most consistent expression profile of all putative effectors identified in this study (adj. *p*-value = 8.37 × 10^−20^) and was systematically over-expressed in PI 88788 virulent nematodes. Characterized as a novel eight-exon gene, Hg8859.1 was determined to encode for a Cathepsin Z-like peptidase protein (Hg-CPZ-1). The coding sequence was confirmed using read coverage-based analysis and via Sanger amplicon sequencing (Table S4). Unlike other differentially expressed putative effectors identified in this analysis, this candidate was the only transcript predicted to be secreted through UPS pathways, with a lack of a traditional secretory signal peptide (prediction < 0.9). Strikingly, this transcript shared high homology (95.7%) with the *H. avenae* putative effector AVA09687.1 (Figure 3a). As for the candidate Hg16414.1, it was predicted to be a variant of the annotated SCN gene Hetgly21698, a membrane-bound lipocalin-related protein. Located on Chromosome 8, Hg16414.1 was found to prematurely terminate after the fourth exon. Sequence analysis identified a putative alternative polyadenylation site located in the fourth intron causing the truncated variant to lose both its transmembrane and its functional lipocalin domain. While no putative function could be identified for the resulting protein, blast analysis showed that it shared some homology (65.0%) with the *H. avenae* putative effector AVA09678.1 (Figure 3b).

## 3. Discussion

In this work, we sequenced the whole transcriptome of individual *H. glycines* nematodes, an approach shown to be useful to study individual nematodes and their effectors [19,28], using the long-read sequencing capabilities of the ONT MinION platform. Although more erroneous in nature, these longer sequences allow for more accurate transcript assembly and quantification, a significant advantage when conducting transcript-level expression analysis [24,29,30]. Moreover, ONT RNA-seq gene quantification has been shown to be comparable to Illumina-based gene quantification, highlighting its potential as a suitable alternative to Next-Generation Sequencing technologies [31]. To our knowledge, this is the first instance where an SMRT-Seq type protocol is combined with long-read sequencing to simultaneously identify novel transcripts and study differential transcript-level expression of effectors within PPNs. Using our approach, shown to be sensitive in its ability to capture 92.4% of all transcripts within our dataset, we mined the transcriptome of virulent and avirulent nematodes to identify novel transcripts putatively involved in virulence. Although we captured most transcripts within our dataset, 7% of transcripts were never observed. These likely represent transcripts expressed in the early stages that were not sampled in the current study.

Differential expression analysis identified 171 upregulated transcripts in PI88788-vir females. While the population of origin was shown to have a marked impact on the PCA distribution of individual samples, likely due to population-specific expression profiles [32], nematode virulence status was shown to be the main force driving the formation of distinct expressional clusters. Interestingly, plant genotype (PI 88788 or Essex) had a lesser impact on gene expression than nematode phenotype. Indeed, clustering of expression similarities showed that even when reared on the susceptible host Essex, some individuals tended to cluster with PI88788-vir nematodes at a rate expected under the population’s female index value against that given resistance source. Therefore, these expression profiles do not seem to be associated with the different environments (plant genotypes) in which the nematodes develop but rather with their virulence profile.

### 3.1. Differential Transcript Expression within Putative Effectors

By contrasting the transcript-level expression of PI88788-vir against Avir females, we uncovered a distinct pattern of effector upregulation consistent with previous findings by our team [19]. This might suggest that virulence against PI 88788 is achieved through the increased expression and secretion of effector proteins, which may reflect the underlined selective pressure to host resistance. In PI 88788, resistance is mediated through copy number variations of the *rhg1-b* allele, with more copies leading to a stronger response due to the increased overall expression of the GmSNAP18, GmAAT, and GmWI12 genes [12,33]. Having coevolved alongside this naturally occurring allele, SCN may have adapted by increasing the expression of its own virulence genes, in a dosage-dependent manner, to counteract the cytotoxic effects caused by the accumulation of abnormal α-SNAP_Rhg1_ proteins within the syncytium [34]; a possibility that has been proposed prior [35]. Additionally, the present analysis highlighted substantially more differential expression of effectors at 18 DAI than previously reported by our team at 14 DAI [19], which could suggest a timing element in the expression of virulence genes. The slow onset of resistance in PI 88788, taking on average 8–10 days before causing complete syncytial cell degeneration, could explain these expressional delays [12]. A more detailed analysis of the temporal expression of effectors, similar to what has been done in the root-knot nematode *Meloidogyne incognita* [36], would be needed to elucidate this possibility. It is worth noting that seven of the eight differentially expressed effectors associated with virulence were located in gene features with a single known transcript suggesting that alternative splicing is not directly involved in virulence. While our data suggest the following, we cannot discount the possibility that some isoforms do exist in effector genes but were not captured in this sequencing. The global lack of downregulated transcripts was somewhat unexpected although in agreement with previously published works [19]. This lack of downregulation may be partially explained by the stringent filtering parameters used in this analysis. While these parameters are meant to ensure that reported differentially expressed transcripts are shared across all three populations of a given phenotype, transcripts lowly downregulated or inconsistently downregulated in one or more populations of a given phenotype will be missed.

### 3.2. Discovery of Novel Effectors Involved in Late-Stage Virulence in PI 88788

Of the eight new effector candidates identified in this analysis, five had a predicted functional domain or associated gene annotation. Among these were two transcripts (Hetgly.04491.t1; Hetgly21677.t1) encoding the nematode-specific family of secreted transthyretin-like protein (TTLs). Across the nematoda phylum, TTL-like proteins are some of the most common secretory proteins observed [37]. While the biological role(s) of TTLs remains largely unknown, functional studies in PPN have suggested their potential involvement in nematode parasitism through their ability to protect and modulate host reactive oxygen species (ROS) activity [38]. For instance, the gland-localized TTL-like effector (MjTTL5) of *M. javanica* was shown to promote host plant ROS-scavenging activity through its interaction with a key enzyme of the ferredoxin/thioredoxin system [39]. A similar mechanism has also been reported in the root-lesion nematode *Pratylenchus penetrans* [40]. Finding two candidate TTLs in our dataset, while not surprising, is intriguing in the context of host–pathogen interaction. Indeed, their late-stage overexpression in PI88788-vir females may highlight an adaptation by virulent nematodes to overcome the toxic effects of ROS produced in greater amounts in PI88788-type hosts [41].

Differential expression analysis also showed significant overexpression of two novel transcripts (Hg8859.1 and Hg16414.1) in PI88788-vir female nematodes. To validate quantitative differences between virulent and avirulent nematodes at the transcript level, qPCR was carried out at three time points (14, 16, and 18 dpi). Expression levels of both transcripts were found to be higher at 18 dpi with noticeably lower expression rates at 16 and 14 dpi. Overexpression by virulent nematodes of both effector candidates, in the later stages of infection, could indicate their potential involvement in the breakdown of resistance and warrant further investigation.

Functional annotation of the candidate effector transcript Hg8859.1 highlighted a C1A peptidase; more specifically a cysteine cathepsin Z-like protein henceforth referred to as Hg-CPZ-1. Cathepsins are a diverse group of hydrolytic proteases with many important physiological and biochemical functions identified thus far [42]. In PPN, several cathepsins (cathepsins B, S, and L) have been implicated in pathogenicity and immune response evasion has been associated with cathepsin L proteases [43,44,45,46,47]. For instance, in the pinewood nematode, *Bursaphelenchus xylophilus*, two cathepsin L proteases (Bx-CAT-1 and Bx-CAT2) were noted to be overexpressed in highly virulent nematode populations and are thought to aid in nutrient uptake and reproducibility [44]. Likewise, another cathepsin L in the root-knot nematode *M. incognita* (Mi-cpl-1) was also shown to be crucial in successful plant–nematode interaction and was found to correlate with parasitic success [48]. However, cathepsin Z proteases, while identified in multiple studies of gland-expressed effectors of PPNs (*H. avenae*—[49]; *Radopholus similis*—[50]; *P. penetrans*—[51]), are seldom reported. To investigate the molecular functions of this candidate, we searched for close homologs in other nematodes. We identified a high homology between this candidate and another putative gland effector identified in *H. avenae* (AVA09687.1). Interestingly, this same effector of *H. avenae* was shown, in a transient expression assay in *Nicotiana benthamiana*, to be capable of suppressing BAX-triggered programmed cell death [49]. Owing to the high degree of similarities between Hg-CPZ-1 and AVA09687.1, we would expect to see similarities in their functions, although functional validations are ongoing. Additionally, it is worth noting that this candidate does share similarities with the characterized *C. elegans* cathepsin Z gene cpz-1 (Ce-CPZ-1). RNA interference studies conducted on this gene found that it was likely involved in cuticle molting, either actively through the proteolysis of cuticle proteins or indirectly through the processing of other proteins involved in cuticle shedding [52]. Supporting this hypothesis, the authors identified an accumulation of native CPZ-1 proteins in the hypodermis/cuticle of larval and adult stages. This was also found to be the case with CPZ-1 in *Onchocerca volvulus* [53]. However, because we observed evidence of expression within gland-cell RNA-seq data and the putative parasitism function observed in a close homolog protein found in *H. avenae*, this would suggest that Hg-CPZ-1 might have acquired an alternative or additional function in SCN. In addition, Hg-CPZ-1 was an interesting example of a putatively secreted, esophageal gland-expressed, cathepsin Z gene. Unlike its closest homolog in *H. avenae* (AVA09687.1), Hg-CPZ-1 did not contain a secretion signal peptide at its N-terminus. However, both SecretomeP and DeepLoc 2.0 analysis predicted its secretion via the UPS pathway, likely through lysosomal exocytosis or an alternative trafficking route [54]. While less common than Golgi-mediated effector secretion [8], some effectors of PPNs have been shown to be secreted via UPS pathways. For example, the recently characterized *M. incognita* effector Mi-ISC-1, a protein shown to interfere with salicylic acid biosynthesis, was identified as an unconventionally secreted effector [55]. Yet, the possibility of effector secretion through UPS pathways has never been fully explored and may warrant further investigation in SCN to better understand the diversity of the effectorome and the mechanism contributing to it.

A protein-coding transcript (Hg16414.1) of the SCN gene Hetgly21698 was also found to be significantly overexpressed in PI88788-vir females. This putative effector was particularly interesting as it was found to be generated through the alternative splicing of a membrane-bound lipocalin-related gene with no associated effector characteristics. Although never documented in PPNs, effectorome diversification through the alternative splicing of a non-effector gene has been observed in other plant pathogens. For instance, in the oomycete *Pseudoperonospora cubensis*, a multidrug transporter gene isoform was noted to generate a functional RxLR-type effector protein (PscRxLR1) [22]. While a more detailed analysis is needed to assess the extent to which alternative splicing-mediated neofunctionalization of non-effector genes occurs, this could constitute an interesting mechanism by which SCN diversifies its effectorome. While this candidate shared some homology with the *H. avenae* putative effector AVA09678.1, no protein function nor conserved sequence domains could be identified. Although finding a pioneer effector gene in SCN is not all that surprising [5,8], a functional analysis study would be needed to assess the function and localization of this candidate.

### 3.3. Genome-Wide Discovery of Novel Coding Transcripts

Generally, while providing valuable insight into the expressed transcripts of an organism, reference transcriptomes often underestimate its true diversity [56]. The difficulty in capturing the full breadth of diversity resides in the fact that many genes, or their given isoforms, are expressed only under specific conditions, in certain tissues, or are unique to some (sub)populations [56]. Yet, systematically discounting these unreferenced transcripts may result in the loss of valuable information. Using our approach, predicated on reannotating the SCN genome using long-read sequencing, we uncovered a significant number of novel transcripts. However, while a marked increase in the reported diversity of the SCN transcriptome, which presently contains 22,465 genes and 1468 isoforms [27], it is by no means a complete characterization. Indeed, in nematodes, it is estimated that between 20% and 30% of all genes undergo some form of alternative splicing [57,58]. For example, in the free-living nematode *C. elegans*, enormous annotation efforts have revealed that just under 28% of the 20,468 genes contain transcript variants (PRJNA13758—[59]). In the plant-parasitic nematode *Globodera rostochiensis*, while less frequent, the latest genomic works have estimated that alternative splicing occurs in just over 15% of all genes (PRJNA695196—[60]). In the present study, we estimate the rate of genes containing transcript variants to be over 11%, a value lower than what has been observed in *C. elegans* and *G. rostochiensis*. One reason for the lower-than-expected rate of alternative splicing may reside in the sampling procedure used in this study. Indeed, we focused our analysis on the J4-stage of infection, chosen to maximize the likelihood of selecting true PI88788-vir females. As such, a time-course experiment would be likely to uncover additional biologically relevant transcripts. Furthermore, traditional SMRT-Seq 2 protocols are known for their five-prime coverage bias, due to inefficient transcription of reads over 4 kb [61]. This bias may have impacted our ability to detect some variants located in the five-prime portion of genes. While our protocol was adapted to limit this possibility, through the use of the SuperScript II reverse transcriptase (RT), a more efficient MMLV-type RT able to generate first strand cDNAs of up to 12.3 kb in length with preferential additions of five-prime non-template cytosine on full-length transcripts, we cannot discount this possibility.

Nevertheless, our approach did prove crucial in the discovery of novel transcripts and novel genes of potential significance in the acquisition of pathogenicity and virulence. Other approaches, such as pool-seq based on short reads, allow the capturing of the full genetic diversity in a population, without having to amplify single nematode transcriptomes but do not allow specific alleles to be associated with traits such as virulence nor to precisely reconstruct the repertoire of splice variants [62].

## 4. Materials and Methods

### 4.1. Plant and SCN Material

Seeds of the soybean lines plant introduction PI 88788 (resistant) and Essex (PI 548667, susceptible), obtained from the USDA Soybean Germplasm Collection, were germinated in humidified Pro-Mix (Premier Tech Horticulture, Delson, QC, Canada) in a 72-plug tray. Trays were placed in a growth chamber set to 28 degrees Celsius (°C) with a 12 h:12 h photoperiod and 50% Relative humidity until they reached the VC stage (~7 days). Three SCN populations from the AAFC collection were selected based on their respective virulence phenotypes against the soybean PI 88788. The female index values (FI) and HG-type of each population selected in this study were validated at the time of the experiment and are presented in Table 1. To validate the HG-type of selected populations, a standard HG-type test was carried out in sand-filled cone-tainers using Essex as the susceptible control [63]. The female index values on PI 88788 were estimated as the ratio between the number of females developing on this resistant variety and the number developing on the susceptible control Essex [63].

### 4.2. Hatching and Selection of Virulent SCN Individuals

To simulate natural hatching conditions, SCN cysts were exposed to soybean root exudates as described by Audette et al. [64]. Briefly, for each of the three SCN populations tested, approximately 1000 cysts were put in a mesh bag (F57, Ankom, Macedon, NY, USA) and placed in a 15 × 15 centimeter (cm) petri dish. Pouches were submersed in a 50:50 solution of tap water and soybean root exudates and maintained at 28 °C for 3 weeks in the dark. The solution was then changed, and freshly hatched J2-staged nematodes were collected over a period of two days and concentrated on a 25 micrometer (µm) diameter sieve. These were then counted and diluted in tap water to form a 500 J2 mL^−1^ inoculum. Three seedlings (VC stage) of both Essex and PI 88788 soybean lines were placed in a 3 cm × 3 cm silicon mold and inoculated by pipetting 1 milliliter (mL) of the SCN inoculum directly onto the roots (three seedlings for each SCN population tested). Roughly 1 cm of moistened sand was added on top of the roots, ensuring sufficient water was added to form a film on the surface of the sand. Plants were maintained in a growth chamber set to 28 °C with a 12 h:12 h photoperiod and 50% RH. After a period of 48 h, the roots were washed free of the inoculation solution and transferred to silicon molds filled with moist sand. Inoculated plants were allowed to grow, under the same conditions as previously stated, for an additional 5 days prior to transferring to a hydroponic system. In this experiment, a hydroponic system was used to obtain unmated females in order to limit gene expression linked to embryonic development and to avoid any genetic contribution from the males.

The hydroponic system consisted of 32-oz Deli containers (Fabri-Kal, Kalamazoo, MI, USA, PK32TC) placed in thick Styrofoam board, with 4.5-inch pre-drilled holes, and placed in a temperature-controlled water table. A 4-mm air supply line (SMC, Noblesville, IN, USA, TU0425) pressurized to 10 PSI was installed beside the containers with T-junctions at every container and capped with an 0.004-inch orifice restrictor (Air Logic, Racine, WY, USA, F2815-161). Inoculated soybean plants were placed through each container lid (one-inch hole) and held in place with rockwool (Figure S2). The containers were filled with 0.75× Hoagland’s No. 2 Basal Salt (Sigma-Aldrich, St. Louis, MO, USA, H2395) solution for 11 days. Plants were maintained at 28 °C with a 16 h:8 h photoperiod and 50% RH. The nutrient solution was filtered daily, to remove any emerging males, and replaced every three days with fresh nutrient solution.

### 4.3. Selection of Nematodes on Differential Plant Genotypes

Eighteen days post-inoculation (dpi), roots from plants were harvested and blended, one at a time, in 300 mL of cold tap water as described by Eisenback [65]. The resulting material was passed through a 250 µm diameter sieve nested on top of a 25 µm diameter sieve. Material from the 25 µm diameter sieve was collected. Single unfertilized fourth-staged juvenile (J4) females were isolated under a stereomicroscope and cleaned of surface contaminants by dipping them in PCR-quality water. A total of six single females developing on Essex and three females developing on PI88788, of comparable developmental stage, were harvested per population tested. The J4-stage was selected for this study to ensure that only individuals possessing the required virulence genes to overcome PI 88788 host-specific R-genes were selected. With the development of avirulent individuals usually being halted at the J3 stage [66], the probability of selecting an avirulent individual on PI 88788 was greatly reduced. Therefore, all the individuals collected on PI 88788 were considered to be virulent regardless of the HG type of their original population. To maximize the chances of obtaining avirulent individuals on the susceptible line Essex, we used populations with low reproduction success (Female Index) on PI-88788, thus containing very few virulent individuals. Still, there was a probability (equal to FI) to obtain virulent nematodes on the susceptible line. To overcome this situation, we included more replicates on this host line and removed the proportion of individuals (similar to FI) that showed a different expression profile.

### 4.4. RNA Extraction

Using a modified version of a nematode-adapted single-cell RNA-seq protocol [19,28,67], RNA was extracted from single females. All steps were carried out as described Ste-Croix et al. [19] with all reagent volumes doubled. To achieve the required concentration and remove excess primers, individual samples were cleaned-up using a 1.2× ratio of Mag-Bind TotalPure NGS bead to PCR product following the manufacturer’s recommended protocol (Omega BioTek, Norcross, GA, USA, M1378-01). The quality of amplified cDNA was assessed with the Agilent 2100 Bioanalyzer using the Agilent DNA 7500 Kit (Agilent Technologies, Santa Clara, CA, USA). Concentration was determined using the dsDNA HS Assay Kit on the Qubit system (Thermo Fisher Scientific, Waltham, MA, USA, Q32854).

### 4.5. Library Preparation

Sequencing libraries for Oxford Nanopore MinION were prepared, using the direct cDNA Sequencing kit (SQK-DSC109) with the Native Barcoding Expansion 1–12 (EXP-NBD104), following the manufacturer recommendations post cDNA synthesis (Oxford Nanopore Technologies, Oxford, UK). A sequencing library consisted of nine individually amplified female transcriptomes (six on Essex and three on PI88788) from the same population. Before multiplexing the final library, barcoded sample quality and average size were assessed with the Agilent 2100 Bioanalyzer using the Agilent DNA 7500 Kit (Agilent Technologies). A total of 20 fmol of each barcoded sample were pooled before adding 5.0 µL of sequencing adapters. The final library was split and sequenced on two R9.4.1 MinION flowcells (Oxford Nanopore Technologies) for a duration of 72 h or to pore exhaustion. A total of three independent libraries, one for each population sampled, were prepared and sequenced in the same fashion.

### 4.6. Read Processing and Novel Transcript Discovery

Sequences were simultaneously basecalled, demultiplexed, trimmed and binned based on their average quality score using Guppy (v6.0.6—Orford Nanopore) with the flags qscore_filtering, min_qscore 8 and barcode_kits EXP-NBD104 set. Then, 30 base pairs were removed on either side of the reads to remove the single-cell primers, using the seqtk trimfq function (v1.2-r94—[68]).

To detect potential novel transcript variants, trimmed reads of individual nematodes were merged and aligned to the current chromosomal assembly of the SCN genome [27] using Minimap2 in splice alignment mode with the parameter -G set to 8k, secondary = no and with genome annotations supplied using the -junc-bed option. The resulting alignment file was converted to a coordinate sorted bam using samtools (v1.9—[69]) and inputted into StringTie2 (v1.5—[70]) with the parameters -L, -f 0.1, -m 75, -c 2, -j 5, -E 25 set. The resulting GFF file was then merged with the current genome annotation file, using the StringTie merge function, and filtered to generate a set of candidate transcripts expressed at a minimum level of 10 transcripts per million (TPM). The bedtools intersect function was then used to filter out single exon candidates, spanning multiple exons of known SCN genes, as these candidates likely represented unmatured mRNA. As an extra filtering step, candidate isoforms predicted in novel gene features were analyzed using RNASamba [71] to filter out transcripts belonging to non-coding genes. Finally, the resulting filtered transcriptome was clustered using cd-hit-est with the parameters c and s set to 0.90 and 0.95, respectively [72]. This step was performed to generate a set of non-redundant transcripts; an important step considering that the genome of SCN is highly repetitive with many gene duplications.

### 4.7. Differential Transcript Expression Analysis

To investigate the differential transcript expression between virulence phenotypes, trimmed reads from individual females were aligned to the StringTie2 filtered sequences using Minimap2 with the parameters -ax map-ont, -N 10, -p 0.99 set (v2.24-r941—[73]). Individual nematode read counts per transcript were extracted from the resulting sam files and merged into a combined count table using the Flair “quantify” module [74]. Read counts were imported into edgeR (V3.28.0—[75]) for differential expression analysis. Samples with less than 100,000 mapped reads were considered of poor sequencing quality and were subsequently removed from the analysis. Transcripts with zero read counts in over 75% of the samples were removed from the analysis. Biological replicates were filtered based on a clustered image map of remaining normalized transcripts counts (Figure S3). Putative avirulent individuals found to cluster with PI 88788 virulent individuals were removed from the downstream analysis as they likely represented a PI 88788 virulent nematode. Being that the Essex cultivar does not apply any selective pressure on developing nematodes, the likelihood of selecting a virulent nematode on this cultivar is equivalent to the population’s female index value against the resistant PI 88788 cultivar. For this same reason, Avir females are likely to contain both avirulent and non-PI 88788 virulent females. Post-filtering, a pairwise contrast of individuals virulent to PI 88788 resistance genes (PI88788-vir) against putative avirulent individuals (Avir) was conducted on a TMM normalized dataset using the ExactTest function. A transcript was deemed differentially expressed when presenting a log_2_ fold-change (log_2_FC) over 2.0 with an adjusted *p*-value under 1.00 × 10^−10^. These stringent parameters were set to ensure that predicted differentially expressed transcripts were shared across all individuals of a given phenotype regardless of the population of origin.

### 4.8. Effector Gene Prediction and Gene Annotation

To identify putative effectors in novel transcripts and differentially expressed transcripts, sequence-based prediction of signal peptide and unconventional protein secretion (UPS) features were carried out, on amino acid sequences, using SignalP-6.0 [76], and SecretomeP-2.0 [77], respectively. With proteins secreted via UPS pathways having been linked to immune response modulation and pathogen virulence, SecretomeP predictions will help gain a more complete picture of the SCN effectorome [78,79]. The TMHMM-2.0 pipeline [80] was then used to predict any transmembrane domain contained within the proteins. A transcript was classified as a putative effector protein when it contained a SignalP value above 0.90 or a SecretomeP value above a 0.75 threshold with no associated transmembrane domain. Subcellular protein localization for each identified differentially expressed transcript was determined using the DeepLoc-2.0 algorithm under default mammalian settings [81]. Finally, the nucleotide sequences of each candidate were compared to the NCBI nr nematodes (taxa: 6231; 1 × 10^−20^ minimum E-value) database using the BLASTx algorithm to obtain annotations. The best alignment score with annotation was conserved and reported. If no annotation was retrieved against the nematode database, the sequences were reannotated against the whole of the NCBI nr database. For this paper, we define a putative effector as a transcript containing either a signal peptide or UPS features, with no transmembrane domain, and predicted to be secreted to the extracellular region of cells.

### 4.9. Validation of Putative Virulence Gene Candidate

Gene validation was carried out via a qPCR approach using the reference housekeeping gene actin (Act-2). This housekeeping gene was determined to be the most stable, out of 22 candidates tested, using the method proposed by Sabeh et al. [82]. Non-related individuals to those sequenced, from the same populations tested as well as a new population sharing similar female index values on PI 88788 to QCSTJ5 and QCSTJ15 (Table 1), were selected on both Essex and PI 88788 soybeans following the same methodology as previously described. To verify product accumulation in response to the gradual onset of host defense mechanisms, female nematodes were harvested at 14, 16 and 18 DAI. A total of eight females per plant genotype, per population, and per timepoint, were harvested and prepared following the above-outlined protocol. For each transcript of interest, qPCR reactions were carried out in duplicates using the QuantiFast SYBR Green PCR Kit (Qiagen). Each reaction contained: 10.0 µL of 2× QuantiFast SYBR Green PCR Master Mix, 5.6 µL of RNase-free water, 1.2 µL each of forward and reverse primer at a stock concentration of 10 µM (Table S4), and 2.0 µL of cDNA at a stock concentration of around 2.5 ng/µL. Real-time quantitative PCR was carried out on the Agilent Stratagen Mx3000P (Agilent Technologies) using the following cycling program: initial heat activation at 95 °C for 5 min followed by 40 cycles of denaturation at 95 °C for 10 s and combined annealing/extension at 60 °C for 30 s. Fluorescence data was captured following each extension cycle. Individual nematode Δct values were grouped, based on plant genotype and harvest date, before being averaged using a geometric mean approach. Relative fold-change (FC) and log_2_FC were estimated using the ΔΔct approach [83]. Samples with no valid ct values for the housekeeping gene were removed from the analysis.

To validate the expression of putative effectors genes within gland-cells of SCN, esophageal gland RNA-Seq data was retrieved from NCBI [9] and reanalyzed using the annotations generated in the present experiment. Briefly, quality-trimmed reads were mapped to the chromosomal genome assembly using the STAR aligner (V2.7.10a—[84]) with the StringTie2 annotations supplied. The Salmon quant (v0.13.1—[85]) module was then used to extract the number of reads corresponding to each transcript model. Transcripts with at least one CPM were considered putatively expressed in gland cells.

## 5. Conclusions

In summary, our results would suggest that, while alternative splicing does occur within effector genes and could lead to the neofunctionalization of non-effector genes, its contribution to SCN virulence is limited. However, a more comprehensive analysis of alternative splicing across the entire life cycle of SCN, and in different sources of resistance, would be needed to fully assess its role. Additionally, while we observed that virulence was most often associated with effector gene upregulation, the underlying genetic mechanisms remain unknown. A study looking further into the genetic differences (epigenetic marks, copy number variation, presence/absence variations) leading to this altered expression state between PI88788-vir and Avir nematodes would help shed light on the question. Moreover, this study showed the advantages of reannotating the genome of SCN under the specific conditions tested. Reannotation of the genome can be helpful in discovering genes and transcripts that may have been missed during the initial annotation process, as many genes are only expressed in specific conditions, populations, or infection stages. This can reveal potentially significant biological information that was previously overlooked.

## Figures and Tables

**Table 1:** Characteristics of selected *Heterodera glycines* populations with their respective female index, origin and HG-type.

Population Name	Original Field Location	Female Index	HG-Type	Experiment
QCSTJ5	Blenheim, Ontario, Canada	16.17.5.0-	1.2-	Sequencing
QCSTJ6	Bothwell, Ontario, Canada	11.6.7.11-	1.4-	Sequencing—qPCR
QCSTJ15	Kingsville, Ontario, Canada	8.18.3.4-	2-	Sequencing—qPCR
QCSTJ-US2	Brown County, Ohio, USA	1.19.-.0-	2-	qPCR

**Table 2:** Partial table of Heterodera glycines putative effector genes with respective SignalP or SecretomeP values and corresponding subcellular localization prediction, gland-cell expression state, and gene annotation values.

Transcript id	Log_2_FC	Adj. *p*-Val.	Localization	SP ^1^	SecP ^2^	GE ^3^	Blastx Annotations	E-Value
Hetgly01325.t1	3.873	7.72 × 10^−13^	Cytoplasm|Nucleus	0.00	0.90	Y	KAI1731729.1 Protein kinase domain-containing protein [Ditylenchus destructor]	1.24 × 10^−152^
Hetgly02290.t1	2.852	2.34 × 10^−12^	Extracellular	1.00	0.70	N	--NA--	--NA--
Hetgly04490.t1	3.243	8.65 × 10^−16^	Lysosome/Vacuole	1.00	0.74	Y	KAF7636717.1 Glyco_hydro_35 domain-containing protein [Meloidogyne graminicola]	0.0
Hetgly04491.t1	4.034	4.20 × 10^−11^	Extracellular	1.00	0.90	Y	CAM84511.1 transthyretin-like protein 2 precursor [Radopholus similis]	7.48 × 10^−61^
Hetgly06729.t1	2.931	9.62 × 10^−14^	Nucleus	0.00	0.81	Y	KAH7714403.1 U1 small nuclear ribonucleoprotein A [Aphelenchus avenae]	4.53 × 10^−93^
Hetgly06908.t1	4.213	5.23 × 10^−13^	Extracellular	1.00	0.69	N	--NA--	--NA--
Hetgly07025.t1	4.347	2.86 × 10^−15^	Mitochondrion	0.00	0.85	Y	KAH7694343.1 Protein MRPL-14 b [Aphelenchus avenae]	9.09 × 10^−62^
Hetgly07480.t1	2.642	2.76 × 10^−11^	Extracellular	1.00	0.87	N	CAD2152915.1 unnamed protein product [Meloidogyne enterolobii]	4.15 × 10^−33^
Hetgly08734.t1	3.360	5.16 × 10^−11^	Extracellular	1.00	0.93	N	--NA--	--NA--
Hetgly08995.t1	2.261	1.95 × 10^−11^	Mitochondrion	0.00	0.76	Y	KAF7638520.1 Acyl carrier protein [Meloidogyne graminicola]	2.08 × 10^−26^
Hetgly09989.t1	2.669	3.05 × 10^−13^	Nucleus	0.00	0.79	Y	--NA--	--NA--
Hetgly10002.t1	2.684	8.59 × 10^−11^	Nucleus	0.00	0.77	Y	KAF7637276.1 RRM domain-containing protein [Meloidogyne graminicola]	2.34 × 10^−119^
Hetgly15196.t1	3.234	1.07 × 10^−13^	Endoplasmic reticulum	1.00	0.48	Y	KAI1725617.1 thioredoxin domain-containing protein [Ditylenchus destructor]	0.0
Hetgly15644.t1	3.647	5.74 × 10^−13^	Peroxisome	0.00	0.78	Y	KAI1705849.1 SCP-2 sterol transfer family domain-containing protein [Ditylenchus destructor]	0.0
Hetgly15709.t1	2.608	1.18 × 10^−11^	Cytoplasm	0.00	0.81	N	KAH7727199.1 catalase B [Aphelenchus avenae]	1.27 × 10^−29^
Hetgly17937.t1	4.284	9.62 × 10^−14^	Cytoplasm	0.00	0.77	Y	--NA--	--NA--
Hetgly18109.t1	4.526	5.09 × 10^−13^	Mitochondrion	0.00	0.91	Y	KHN88347.1 28S ribosomal protein S18a, mitochondrial [Toxocara canis]	3.00 × 10^−60^
Hetgly18314.t1	3.235	1.14 × 10^−11^	Cytoplasm	0.00	0.79	Y	KAH7727561.1 60S ribosomal protein L14 [Aphelenchus avenae]	2.41 × 10^−58^
Hetgly20098.t1	3.274	7.98 × 10^−11^	Cytoplasm	0.00	0.82	Y	KAH7730990.1 60S ribosomal protein L34 [Aphelenchus avenae]	2.39 × 10^−52^
Hetgly20289.t1	3.348	2.29 × 10^−11^	Cytoplasm	0.00	0.81	Y	KAI1728922.1 ribosomal protein s19e domain-containing protein [Ditylenchus destructor]	1.00 × 10^−70^
Hetgly21523.t1	4.403	8.52 × 10^−12^	Endoplasmic reticulum	0.00	0.93	N	--NA--	--NA--
Hetgly21677.t1	4.491	2.17 × 10^−12^	Extracellular	1.00	0.43	N	KAF7634869.1 AAA domain-containing protein [Meloidogyne graminicola]	1.02 × 10^−41^
Hetgly21799.t1	3.238	1.63 × 10^−11^	Nucleus	0.00	0.75	Y	CAD2202259.1 unnamed protein product [Meloidogyne enterolobii]	9.50 × 10^−44^
Hg11990.1	2.169	5.19 × 10^−11^	Endoplasmic reticulum	1.00	0.30	Y	KAI1725554.1 hsp70 protein domain-containing protein [Ditylenchus destructor]	2.11 × 10^−139^
Hg11990.3	2.874	6.06 × 10^−11^	Endoplasmic reticulum	1.00	0.30	Y	KAI1725554.1 hsp70 protein domain-containing protein [Ditylenchus destructor]	1.36 × 10^−40^
Hg16414.1	3.474	2.76 × 10^−11^	Extracellular	0.97	0.69	Y	AVA09678.1 putative effector protein [Heterodera avenae]	6.27 × 10^−117^
Hg17279.2	3.423	1.01 × 10^−11^	Cytoplasm|Nucleus	0.00	0.82	Y	KAI1717340.1 ribosomal protein l21e domain-containing protein [Ditylenchus destructor]	2.49 × 10^−91^
Hg17540.2	3.303	3.95 × 10^−11^	Nucleus	0.00	0.77	Y	KAF7639258.1 40S ribosomal protein S6, partial [Meloidogyne graminicola]	3.31 × 10^−140^
Hg4745.1	3.307	1.36 × 10^−13^	Cell membrane	0.00	0.88	Y	--NA--	--NA--
Hg835.1	2.835	3.88 × 10^−12^	Nucleus	0.00	0.76	N	KAI1727318.1 CAF1 family ribonuclease domain-containing protein [Ditylenchus destructor]	8.24 × 10^−43^
Hg8859.1	4.145	8.37 × 10^−20^	Extracellular	0.00	0.76	Y	AVA09687.1 putative effector protein [Heterodera avenae]	0.0

^1^ SP represents SignalP prediction values. ^2^ SecP represents SecretomeP values for unconventional secretion feature predictions. ^3^ GE represents the status of gland expression where “Y” is Yes and “N” is No.

## Data Availability

The raw sequences supporting these findings are available under the Bioproject PRJNA956871.

## Supplementary Materials

The supporting information can be downloaded at: https://www.mdpi.com/article/10.3390/ijms24119440/s1.
