# Supplementary material for: Single Nematode Transcriptomic Analysis, Using Long-Read Technology, Reveals Two Novel Virulence Gene Candidates in the Soybean Cyst Nematode, Heterodera glycines
Source: Int J Mol Sci. 2023 May 29;24(11):9440. doi: 10.3390/ijms24119440 (PMC10253548; doi:10.3390/ijms24119440)
Supplement: Supplementary file 1 [file ijms-24-09440-s001.zip › Figure S2.pdf]

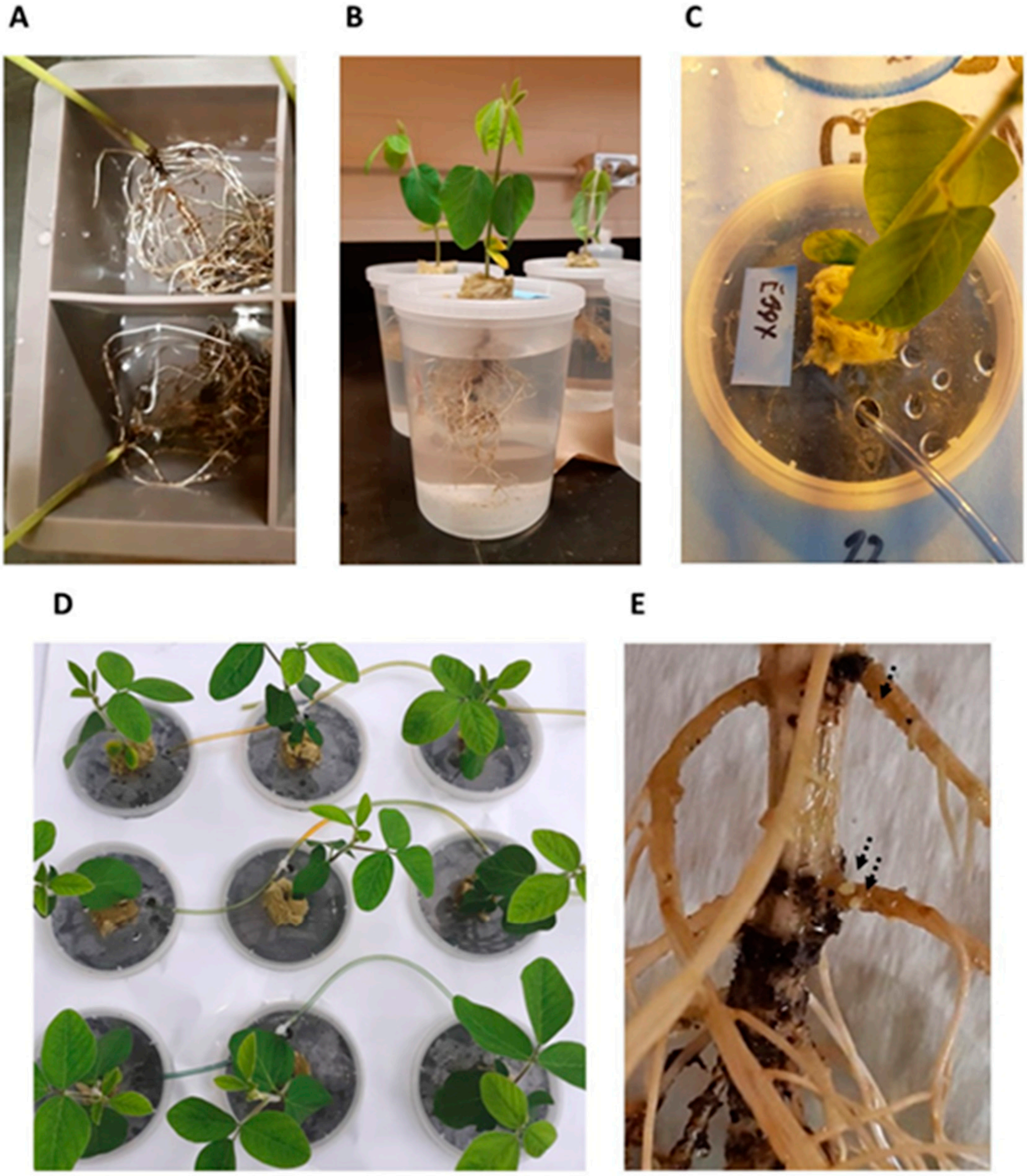

Figure S2: Picture of an (A) submersed inoculation cell. a (B) Hoagland solution-filled container with inoculated plant ready for the hydroponic system. a (C) close-up of rock wool plant support in the container lid and a (D) general view of containers in the Styrofoam board floating in the water table. (E) Indicated by arrows are individual females of *Heterodera glycines* visible at the surface of the roots 18 DAI.
