# Supplementary material for: Single Nematode Transcriptomic Analysis, Using Long-Read Technology, Reveals Two Novel Virulence Gene Candidates in the Soybean Cyst Nematode, Heterodera glycines
Source: Int J Mol Sci. 2023 May 29;24(11):9440. doi: 10.3390/ijms24119440 (PMC10253548; doi:10.3390/ijms24119440)
Supplement: Supplementary file 1 [file ijms-24-09440-s001.zip › Figure S3.pdf]

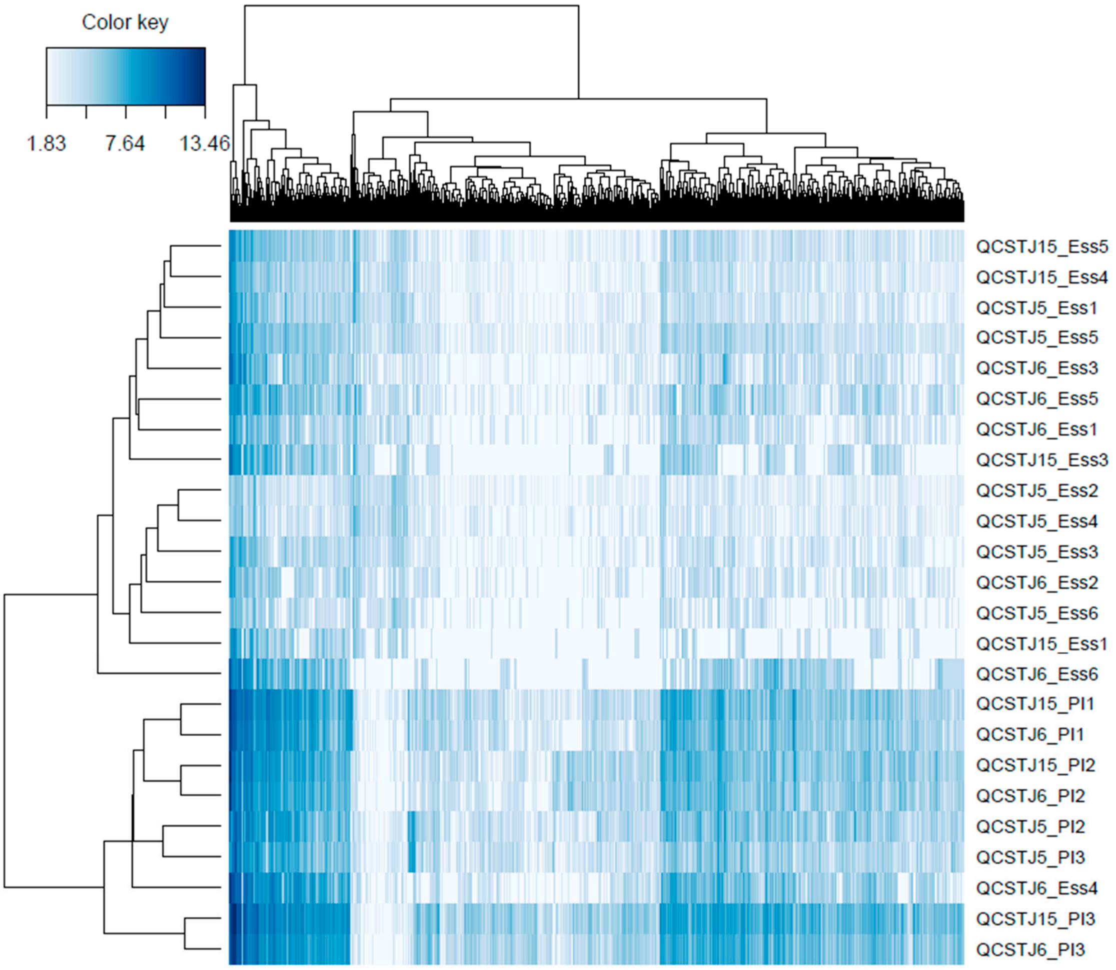

Figure S3: Clustered image map of normalized transcripts counts with expressional clustering of individual *Heterodera glycines* nematode transcriptomic profiles
